# RASAL2 suppresses the proliferative and invasive ability of PC3 prostate cancer cells

**DOI:** 10.18632/oncotarget.28158

**Published:** 2021-12-21

**Authors:** Krishma Tailor, Joseph Paul, Somiranjan Ghosh, Namita Kumari, Bernard Kwabi-Addo

**Affiliations:** ^1^Department of Biochemistry and Molecular Biology, Howard University, Washington, DC 20059, USA; ^2^Department of Biology, Howard University, Washington, DC 20059, USA; ^3^Center for Sickle Cell Disease, Howard University, Washington, DC 20059, USA

**Keywords:** RASAL2, Ras-protooncogene, prostate cancer, TNFα signal, DNA methylation

## Abstract

The RAS protein activator like 2 (RASAL2) negatively regulates RAS proto-oncogene which is activated by high mutation rate in cancer. Thus, RASAL2 expression could potentially limit the function of RAS in prostate cancer (PCa). Genome-wide DNA methylation analysis demonstrated that RASAL2 is differentially hypermethylated in PCa tissues compared to benign prostate tissues. The PCR analysis of RASAL2 mRNA transcript showed differential expression in a panel of prostate cell lines with most PCa showing lower RASAL2 expression compared to benign prostatic epithelial cells. In PCa PC3 cells, the ectopic expression of RASAL2 significantly inhibited cell proliferation and invasion and induced an S phase plus G2/M phase cell cycle arrest. Ingenuity Pathway Analysis (IPA) demonstrated a cross talk between RASAL2 and TNFα, a key cytokine in immune signaling pathway that is relevant in PCa. Over-expression of RASAL2 downregulated TNFα expression whereas the knockdown of RASAL2 caused increased expression of TNFα. Taken together, our data demonstrates tumor suppressor role for RASAL2 in human PCa cells, despite increased RAS oncogenic activity. Our observation provides a new mechanistic insight of RASAL2 expression in aberrant Ras expression and immune signaling in PCa cells suggesting a potential novel therapeutic target for PCa.

## INTRODUCTION

Prostate cancer (PCa) is the second leading cause of cancer-related death in the United States. In 2021, the American Cancer Society estimated 2,48,530 new PCa cases, and this is accompanied by 34,130 deaths [1]. The well-established risk factors for PCa are increasing age, African ancestry, and family history of the disease. There is considerable racial disparities with regards to prostate cancer risks. African America (AA) men demonstrates 60% higher PCa incidence compared to European American (EA) men [2]. A complex combination of socioeconomic factors and lifestyle/environmental exposure contributes to PCa disparity [3–5] and biological factors may also play a significant role in PCa disparity [3, 4] whereby differential genetic and epigenetic alterations may account for higher PCa incidence and mortality rate in AA men compared to EA men. A number of genetic and epigenetic alterations are associated with PCa suggesting that there is not a single predominant genetic pathway that is associated with the disease etiology and/or progression [6]. Androgen biosynthesis and androgen receptor (AR)-mediated signaling pathway [7], PTEN (phosphatase and tensin homolog), a negative regulator of PI3K/AKT signaling pathway [8], and p53 (tumor suppressor protein 53) genes [9, 10], RAS signaling pathway [11] are differentially altered in PCa patients belonging to one racial/ethnic group compared to other groups.

The Ras pathway is one of the most studied and frequently dys-regulated in human cancers [12]. RAS alterations contribute to 20–30% of all human cancers [13]. Mutations in *RAS* genes occur in a variety of tumor types [14, 15]; however, the Ras pathway is also frequently activated as a consequence of alterations in upstream regulators and downstream effectors, underscoring the importance of this pathway in cancer [12]. Ras is negatively regulated by Ras *G*TPase-*a*ctivating *p*roteins (RasGAPs), which catalyze the hydrolysis of Ras-GTP to Ras- GDP [16]. As such, RasGAPs are poised to function as potential tumor suppressors. RAS protein activator like 2 (RASAL2) is a member of the family of RAS GTPase-activating proteins (GAP). The RASAL2 protein negatively regulates the RAS signaling pathway by catalyzing the hydrolysis of RAS-GTP to RAS-GDP in many cellular activities and acts as a vital regulator of the RAS signaling pathway. In triple negative breast cancers [17, 18], liver cancer [19, 20] and colorectal cancer [21] RASAL2 has been observed to promote carcinogenesis. On the other hand, tumor suppressor role has been described for RASAL2 in a number of cancer types including luminal B breast cancers [22–24]; renal cancers [25]; bladder cancer [26, 27]; astrocytoma’s [28]; nasopharyngeal cancer [29]; pancreatic cancer [30]; ovarian cancer [31] and lung cancer [32]. These studies indicate that the biological function of RASAL2 is influenced by cellular context to influence its pro or anti-oncogenic activity in human cancers [25] and different signaling pathways (as well as cross-talk) of RAS signaling pathway, the RAS-ERK pathway, phosphoinositide-3-kinase (PI3K)/AKT/mechanistic target of rapamycin (mTOR) signaling pathway, and nuclear factor (NF)-κB pathway may account for the different biological outcomes of RASAL2 activity. The *RASAL*2 gene is commonly inactivated by epigenetic mechanism in malignant tumors [13]. However, there is little information on the biological role of RASAL2 in human PCa.

In the present study, we have identified *RASAL*2 to be differentially hypermethylated in PCa compared to benign prostate tissues in a Genome-wide DNA methylation analysis by Devaney et al. [33]. Additionally, Ingenuity Pathway Analysis [34] suggested a crosstalk between RASAL2 and tumor necrosis factor alpha (TNFα) in prostate cancer. The TNFα is a pro-inflammatory molecule that may play an important role in the development of the immune response and affect the progression of PCa [35] and several studies have shown differential inflammatory microenvironment in PCa patients and may also underlie disproportionate incidence and unfavorable outcomes in AA men [36]. Mechanistically, a crosstalk between TNFα and RASAL2 in PCa may provide a new insight into RASAL2 signal transduction in modulating the tumor microenvironment. We demonstrate that RASAL2 functions as a suppressor of PCa cell proliferation and invasive ability and may alter the immune microenvironment of PCa cells that may contribute to PCa disparity.

## RESULTS

### Genome-wide DNA methylation of RASAL2 in prostate tissues

**Figure 1:**
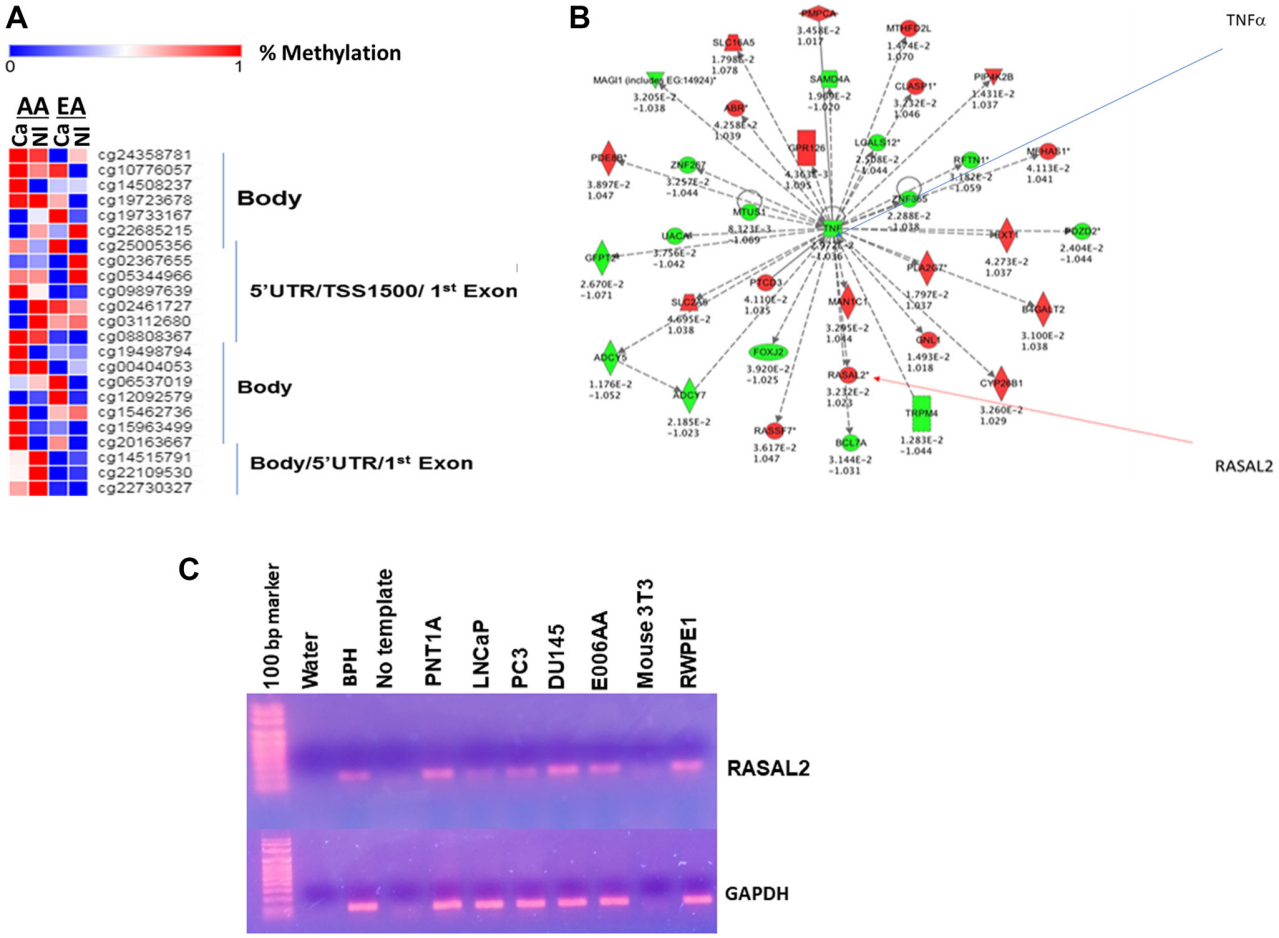
(**A**) Heat map hierarchical cluster analysis of CpG probesets in RASAL2 genome identified in a panel of prostate tissues (used in genome-wide DNA methylation analysis [33]). African American (AA) benign tissues (Nl) and AA cancer tissues (CA); European American (CAU)-Nl and CA. (**B**) Ingenuity Pathway Analysis of TNFα and RASAL2 crosstalk show a crosstalk between TNFα and RASAL2 (*p* value < 0.05). (**C**) Expression of RASAL2 and GAPDH by PCR analysis. Negative controls: water, no template, mouse 3T3 cells. Benign prostate cell lines: PNT1A and RWPE1. Prostate cancer cells: LNCaP, PC3 and DU145. Kidney cancer cell line; E006AA.

In order to assess the methylation status of RASAL2 gene in prostate tissues, we queried the genome-wide DNA methylation data [33] and identified all the CpG probesets in PCa tissues and benign prostate tissues from AA and EA patient samples used in the methylation analysis. The data showed differential methylation of the RASAL2 gene in the entire genome of tissue samples analyzed (Figure 1A). We observed hypermethylation of many CpG probes in cancer samples compared to benign tissues in both AA and EA tissues. We did not observe any data of differential RASAL2 gene expression in AA versus EA samples in the TCGA database (results not shown). Overall, our data showed higher differential methylation in the AA PCa and prostate tissue samples compared to the EA samples. Ingenuity Pathway analysis showed crosstalk of RASAL2 and several genes including TNFα which is an important signaling intermediate in prostate carcinogenesis (Figure 1B). Furthermore, RASAL2 expression at the RNA transcript level showed differential expression in a panel of prostate cell lines with PCa cell lines showing lower expression compared to the primary immortalized prostate cells (Figure 1C). The normal kidney epithelial cell line (E006AA) was used as a positive control, as previous report suggests high expression of RASAL2 in immortalized kidney epithelial cell [25], whereas the mouse embryonic fibroblast cells (mouse 3T3) was used as a negative control) (Figure 1C). Our results demonstrate differential hypermethylation of RASAL2 in prostate tissues and could be a potential mechanism for the regulation of RASAL2 expression in PCa and PCa disparity.

### Functional analysis of RASAL2 expression and signal targets in prostate cancer

**Figure 2:**
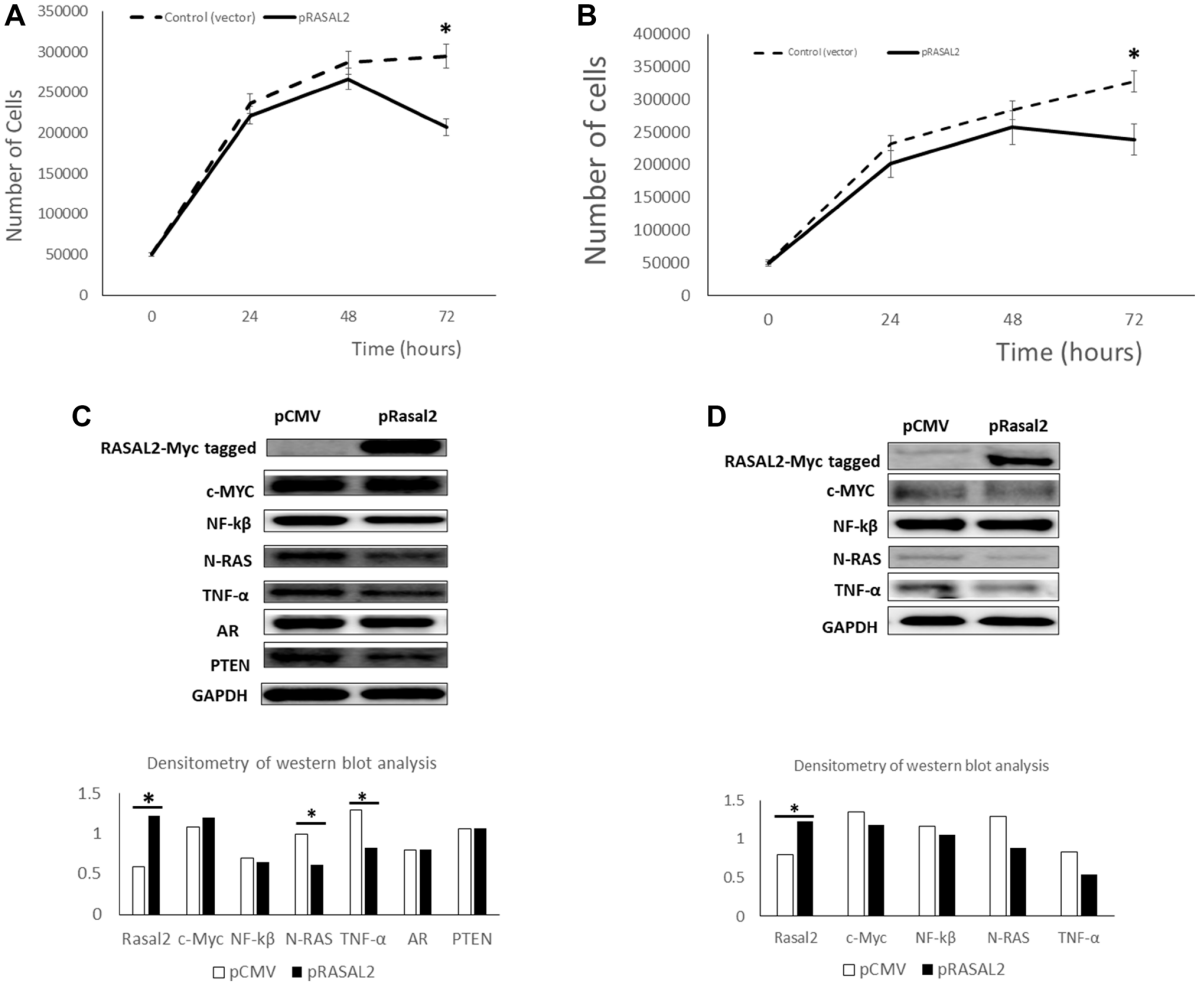
Transient transfection of RASAL2 in LNCaP and PC3 prostate cancer cells. (**A**) The LNCaP PCa cell line was transfected with a RASAL2 cDNA cloned in the mammalian expression vector (pCMV-Myc-DDK) or the pCMV vector only. At the indicated times after transfection, cells were trypsinized and counted using a Coulter counter. Similar transfections were done in PC3 PCa cell line (**B)**. (**C**) Protein extracts were collected from LNCaP cells at day 3 after transfection with RASAL2 expression plasmid or empty vector and analyzed by Western blotting with the following antibodies; RASAL2-Myc tagged antibody, C-myc, NF-kB, N-RAS, TNFα, AR, PTEN and GAPDH. Densitometry of western blot analysis is shown as the ratio of each protein expression to GAPDH protein. Similar experiments were carried out in PC3 cells (**D**). Statistical significance is indicated as (^*^ *p* < 0.05; *t*-test). Data shown are representative of 3 independent experiments.

To ascertain the biological function of RASAL2 in PCa, the LNCaP (androgen-dependent) and PC3 (androgen-independent) cells we transfected pCMV6–RASAL2 (encoding the full length of Rasal2 complete ORF with an expression Myc-DDK tag) or pCMV empty vector as a negative control (Figure 2A and 2B). We chose LNCaP and PC3 cells because these two cell lines expressed relatively low amount of RASAL2 mRNA transcript compared the primary immortalized prostate cells and DU145 (shown in Figure 1C). The transient transfection of RASAL2 expression vector after 72 hours showed significant inhibition of cell proliferation in LNCaP cells (30% reduction) and PC3 cells (25% reduction) when compared to the control (empty vector). To determine if the transient transfections altered the protein expression of RASAL2 and signaling we carried out Western blot analysis (Figure 2C and 2D). The results showed significant increased expression of RASAL2 protein in the LNCaP transiently transfected cells compared to the pCMV-vector only transfection (Figure 2C). This was accompanied by significant reduction in N-RAS and TNFα in response to increase expression of RASAL2 protein (Figure 2C). On the other hand, we did not see significant changes in PTEN, c-Myc, NF-_k_B and AR expression in transiently transfected cells with RASAL2 compared to the empty vector only in LNCaP cells (Figure 2C). Similar observations were made in PC3 cells transiently transfected with RASAL2 expression vector compared to the empty vector transfection (Figure 2D). We observed significant high expression of RASAL2 and decrease expression of N-RAS, and TNFα (not significant) and a modest change in NF-_k_B expression in the PC3 cells transiently transfected with the RASAL2 vector compared to the empty vector only transfection (Figure 2D). The data demonstrates the increased expression of RASAL2 in PCa cells inhibits cell proliferation and this is associated with reduction in N-RAS and TNF-a, but not AR expression suggesting that RASAL2 activity in PCa cells is independent of AR signaling.

**Figure 3:**
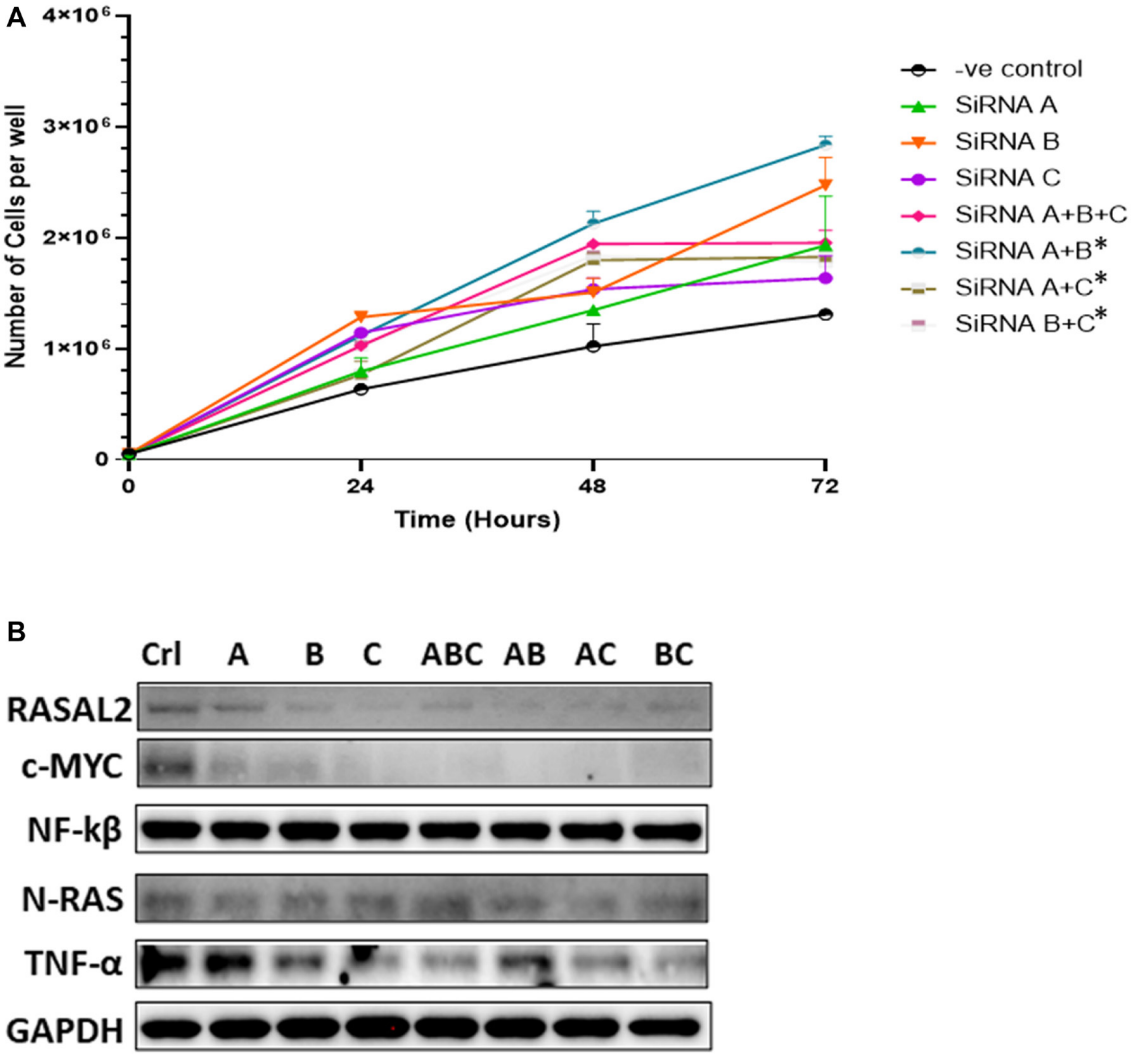
(**A**) siRNA-mediated knockdown transfections. PC3 cells were transiently transfected with one of three different RASAL2-specific siRNA or in combinations, or with a scramble nonsense siRNA (negative control). At the indicated times after transfection, cells were trypsinized and counted using a Coulter counter. Data shown are representative of 3 independent experiments. (**B**) Protein extracts were collected from PC3 cells at the day 3 after transfection with the siRNAs and analyzed by Western blotting with the following antibodies: RASAL2-Myc tagged antibody, C-myc, NF-kB, N-RAS, TNFα, AR, PTEN and GAPDH. Significant difference in cells transfected with RASAL2-specific siRNAs compared to negative control (scrambled siRNA; -ve control) using Fisher *t*-test (statistical significance is shown as ^*^ *P* < 0.05). Data shown are representative of 3 independent experiments.

One of the functional consequences of DNA hypermethylation is silencing gene expression. To mimic gene silencing, we transiently transfected siRNA targeted to RASAL2 in PC3 cells and assessed cell proliferation (Figure 3A). We observed increased cell proliferation in response to different siRNA subunit and the combination of different siRNAs compared to the scramble control, whereby we observed significant increase in cell proliferation for siRNA (A+B; A+C and B+C) at the 72-hour post-transfection. Analysis of protein expression showed that siRNAs caused decreased expression of RASAL2 and c-Myc compared to the scrambled control and this was more pronounced when different siRNA subunits were combined (Figure 3B). The siRNA knockdown also resulted in differential expression of TNFα and N-RAS compared to the scrambled control with some siRNA showing higher expression whereas others showed lower expression compared to the control. Overall, the gene knockdown analysis showed increased cell proliferation, and this was associated with decrease expression of RASAL2, and c-Myc but modest increased in N-RAS expression. Taken together, our observation indicates that RASAL2 expression may play a role in PCa cell proliferation.

### RASAL2 expression and cell migration and invasion in PC3 cells

**Figure 4:**
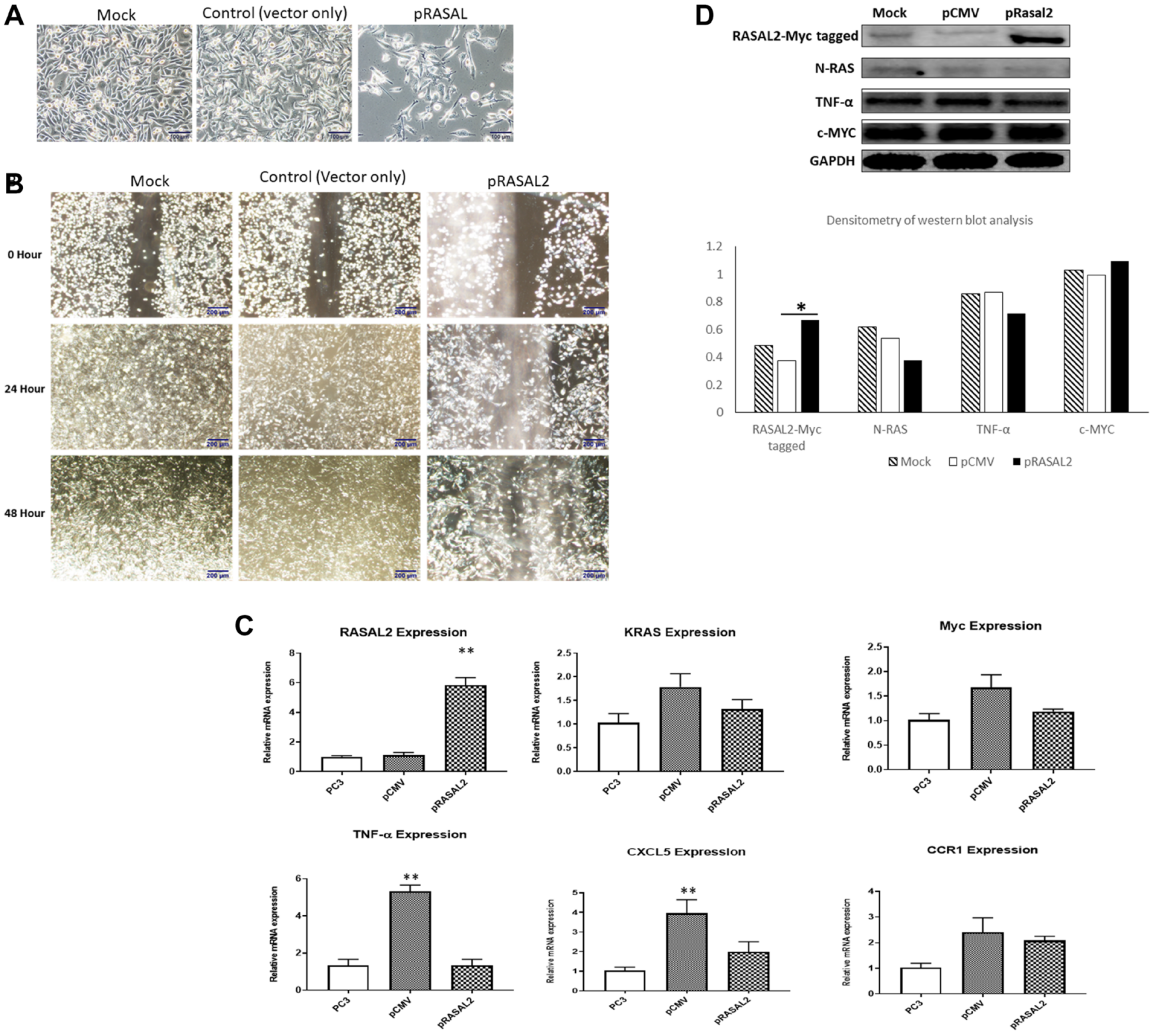
(**A**) Cell growth and morphology of stably transfected PC3 with RASAL2 vector or empty vector compared to untransfected cells. (**B**) Wounding assay of scatter/migration. PC3 cells stably transfected with RASAL2 plasmid or vector only were used in a scratch wound assay as described in Materials and Methods. The cells were permitted to migrate to the area of clearing for a total of 48 hrs and photomicrographs taken at 0, 24 and 48 hrs. Results shown are typical of 3 separate experiments. (**C**) qRT-PCR of RASAL2 expression in stably transfected cells (**D**) Western blot analysis of PC3 cells stably transfected with RASAL2 vector or empty vector only. Densitometry of western blot analysis is shown as the ratio of each protein expression to GAPDH protein. Statistical significance is indicated as ^*^(*p* < 0.05; *t*-test). Data shown are representative of 3 independent experiments.

Increased cell migration and invasion is one of the characteristics associated with highly malignant phenotype of PCa therefore we investigated RASAL2 expression in PC3 cells, which is a highly migratory, invasive, and aggressive PCa cell line [37]. To ascertain the biological effect of RASAL2 on PC3 cell migration and invasion, the RASAL2 expression plasmid was transfected into PC3 cells, and transfected cells selected in G418. The stable overexpression of RASAL2 in PC3 cells demonstrated slow growth phenotype and change in cell morphology compared to empty vector and mock (Figure 4A). To evaluate the effect of over-expressing RASAL2 on PC3 cell migration, we utilized the scratch wound assay be assessing the rate of wound closure after scraping cells from an area of monolayer cultures (Figure 4B). Confluent PC3 cells were scraped, and cells were allowed to migrate for 48 hrs. As shown in Figure 4B, control cells which were G418-resistant but not over-expressing RASAL2 (pCMV-vector only; control transfection) demonstrated higher rates of migration or wound closure when compared to cells over-expressing RASAL2, which showed slower closure rate at the 24 hr and 48-hr time points. This experiment was replicated a total of three times with identical results. We validated RASAL2 expression at the RNA transcript level by qRT-PCR (Figure 4C). The results of qRT-PCR analysis showed 4-fold increase of RASAL2 expression in the RASAL2 stably transfected PC3 cells compared to the empty vector control or mock control. We observed a modest decrease of RAS and Myc expression in the cells transfected with pRASAL2 plasmid compared to the empty vector transfection. On the other hand, we observed a significant reduction in TNFα in pRASAL2 transfected cells compared to the vector only transfection. The mRNA expression of RAS and TNFα were decreased in PC3 transfected with pRASAL2 compared to empty vector pCMV and mock control (Figure 4C). We performed qRT-PCR of the proinflammatory chemokines CXCl5 and CCR1 [38, 39]. The result showed more than 2-fold decrease in CXCl5 expression and a modest decrease in CCR1 expression in PC3 cells transfected with RASAL2 plasmid compared to empty vector transfection (Figure 4C). Western blot analysis confirmed significant high expression of RASAL2 in the stably transfected PC3 cells and a modest reduction in RAS and TNFα proteins (Figure 4D) compared to the empty vector control or the mock untransfected PC3 control.

**Figure 5:**
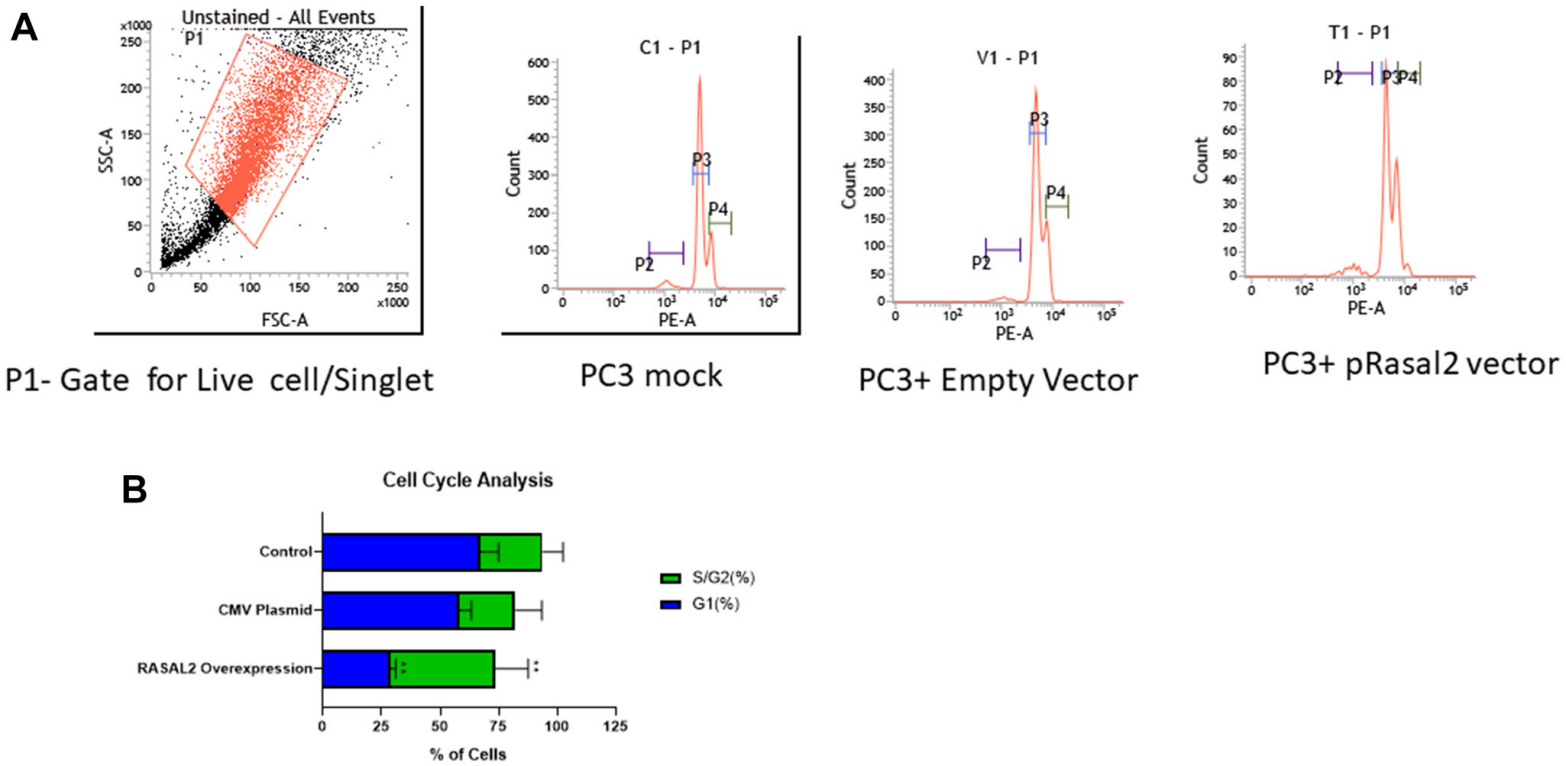
(**A**) Flow cytometry analysis on cell cycle in PC3 cells stably transfected with RASAL2 vector or empty vector only. Representative figure and percentage of cells in each phase of cell cycle. (**B**) Histogram of cell cycle analysis in PC3 stably transfected with RASAL2 plasmid or empty vector compared to mock untransfected cells. P2- gate of apoptotic cells; P3- gate for G1 phase and P4- gate for S/G2 phase of the cell cycle. Data shown are representative of 3 independent experiments.

Finally, we examined the role of RASAL2 in PC3 cell cycle transformation. Flow cytometry showed that the over-expression of RASAL2 inhibited cell growth through inducing cell cycle arrest in S plus G2/M phase as compared to empty vector and mock (Figure 5A and 5B). The results indicated that the number of cells in the S plus G2/M phase were markedly increased in the pRASAL2, compared with the empty vector control and Mock PC3.

## DISCUSSION

One important way that cancer cells grow uncontrollably is by expressing mutations in *RAS* genes and converting them to become oncogenic. Cancer cells may exhibit dysregulation of RAS signaling pathways by alterations in the upstream regulators and downstream effectors. It is estimated that RAS genes exhibit gain-of-function point mutations in 25% to 30% of tumors [40]. Activation of RAS proteins stimulates multiple signaling cascade that control metabolism, proliferation, and transformation. Two best characterized effector pathways of the RAS proteins are the RAF-MEK-ERK and PI3K-AKT pathways [41]. The first downstream effector of RAS identified was the Serine/Threonine kinase RAF. Activation of RAF initiates the RAF-MEK-ERK kinase cascade. Additionally, RAS can activate PI3K and promote PI3K-AKT signaling. AKT, a known protein kinase B (PKB) phosphorylates and inhibit Bad, thus derepressing the anti-apoptotic proteins Bcl-2 and Bcl-XL. AKT also activates IkB kinase (IKK) which in turn phosphorylate IkB, thus resulting in the de-repression of the anti-apoptotic transcription factor NF-kB. Translocation of NF-kB to the nucleus activates genes which promotes cell survival, migration, and epithelial-to-mesenchymal transition (EMT). In addition to activating mutations in RAS protooncogene, the loss of function of negative regulators of RAS, namely the Ras GTPase-activating proteins (RasGAPs) is common in cancer. Of the RasGAPs, RASAL2 is the least characterized and its importance in human disease has only recently begun to be appreciated. Traditionally, RASAL2 functions as a tumor suppressor in lung cancer, ovarian cancer, pancreatic cancer, bladder cancer and luminal B breast cancer [42]. Yet, other studies have reported RASAL2 to have oncogenic activity in triple negative breast cancer and human hepatocellular carcinoma [42]. For instance, RASAL2 expression is frequently suppressed in luminal B breast tumor cell lines and in primary tumors [22, 43]. Nearly 25% of luminal B tumors, the most aggressive form of breast cancers exhibits a concomitant loss of RASAL2. Furthermore, RASAL2 cooperates with other RasGAPs to suppress transformation and metastasis [43] indicating tumor suppressor function for RASAL2 in breast cancer. However, one report has characterized RASAL2 as oncoprotein in triple negative breast cancer [18]. It is believed that RASAL2 can promote cancer progression by activating the oncoprotein RAC1, a RAS-related small GTPase. This effect of RASAL2 is dependent on the EMT status of a cell. These observations have raised the issue as to whether RASAL2 functions as a tumor suppressor or oncoprotein. Yet, these opposing conclusions may be the results of functional complexity, and cancer cellular context, the EMT status of a cell and the heterogeneity of molecular cancer pathways. Further investigations are needed to resolve the conflicting reports of RASAL2 activity in tumorigenesis. In the present study, we have found that RASAL2 protein may have an important role in controlling RAS biological activity in PCa cells. RASAL2 is hypermethylated in PCa in comparison to normal prostate tissues. Differential hypermethylation in AA PCa compared to EA suggests that disproportionate frequency of hypermethylation and gene silencing of RASAL2 in AA PCa could affect the disease milieu and contribute to the aggressive PCa observed in AA men. In our analysis, we have found RASAL2 to suppress cell proliferation in *in-vitro* LNCAP and PC3 cells. In addition, RASAL2 inhibited cell migration and invasiveness as well as cell cycle arrest in S and G2 phase. Our observation is consistent with previous report of tumor suppressor function for RASAL2 in breast cancer [22] and colorectal cancer [44]. In PCa cell lines we have found that RASAL2 function appears to be independent of androgen or PTEN signaling. On the other hand, RASAL2 could potentially interact with immune regulators such as TNFα [45] and regulate other TNFα mediated downstream pro-inflammatory cytokines, as demonstrated in our analysis whereby the increased expression of RASAL2 did not cause significant decrease in TNFα expression but also one downstream effector; CXCL5. TNFα pro-inflammatory activities induces cell proliferation, differentiation, and apoptosis and TNFα function are achieved through activating multiple signaling pathways, especially the activation of transcription factor NF-kB. Interestingly, some studies have indicated that TNFα cooperates with RAS in promoting metastasis in at least breast and lung cancers [46, 47]. In our study, it appears that there is a crosstalk between RASAL2 and TNFα signals in prostate carcinogenesis. Thus, the ability of RASAL2 to target RAS and TNFα signal and inhibit the proliferative and invasive capacity of PCa cells indicates that restoration of RASAL2 expression or the use of synthetic or small molecule that can replace RASAL2 may be an attractive novel therapeutic approach that will not only target RAS oncogenic activity but can also immune signals in aggressive PCa treatment. Future studies to investigate RASAL2 expression in PCa tissues and examine relationship with clinical cancer stage as well as histological grade are warranted as RASAL2 has the potential as a novel prognostic marker and a therapeutic target for PCa and other cancers. In conclusion, the present study demonstrates that overexpression of RASAL2 inhibits PCa cell proliferation, cell migration, and decrease the RAS protooncogene protein, thereby altering RAS pathway, as well as decreasing the TNFα protein proinflammatory master cytokine.

## MATERIALS AND METHODS

### Cell culture

The human PCa cell lines; LNCaP (androgen-dependent) and PC3 (androgen-independent) were obtained from the American Type Culture Collection (ATCC). These cell lines were maintained in RPMI-1640 supplemented with 10% fetal bovine serum (Gibco), 100 ug/ml streptomycin and 100 U/ml Penicillin (Cellagro), unless otherwise indicated. All cell lines were cultured in a humidified 5% CO_2_ air atmosphere at 37°C.

### Cell transfection

For over-expression of RASAL2, the LNCaP and PC3 cells were plated at 0.5 × 10^5^ cells/well in a 24-well plate and transfected with 0.5 ug/well of *RASAL*2 plasmid construct pCMV-Rasal2 Myc-DDK tagged (OriGene- Cat. No. -RC223449) or empty pCMV vector (origene) using Lipofectamine LTX transfection reagent (Invitrogen) and according to the manufacturers protocol. After 24, 48, or 72 hours (hrs) post-transfection, cells were trypsinized and counted using a Coulter counter. To establish stably transfected cells, two days post transfection, cells were selected in Geneticin (Gibco) containing medium at a final concentration of 500 ug/ ml. After 14 days into the selection Geneticin resistant clones were pooled together and propagated. A second transfected plate was used for RNA and Protein extraction for quantitative-RT-PCR and western blot analysis respectively. Knock-down of RASAL2 was carried out by transient siRNA (OriGene- Cat No. SR306275) transfection. The PC3 and LNCaP cells were plated at 0.5 × 10^5^ cells/well in 24 wells plate and transfected with individual siRNA or combination of siRNA duplex or scrambled negative control siRNA duplex using Lipofectamine-RNAiMax transfection reagent (Invitrogen) according to the manufacturers protocol. After 24-, 48-, or 72-hrs post-transfection, cells were trypsinized and counted using a Coulter counter. A second transfected plate was used for RNA and protein extraction.

### Western blotting

Cells were harvested and lysed in radioimmune precipitation assay (RIPA) buffer, 0.5M EDTA and proteases and phosphatase inhibitors cocktail total protein quantified by BCA protein assay kit (Pierce). For Western blots, 30 mg of protein extract/lane were electrophoresed, transferred to nitrocellulose membrane (Invitrogen) and incubated overnight with each of the following primary mouse monoclonal antibody: RASAL2 (1:2000; sc-390605; Lot# 2516); NF-kB (1:500; sc-8008; Lot# H1220); N-RAS (1:1000; sc-31; Lot# JQ520); TNFα (1:500; sc-515766 Lot# 17020); C-myc (1:500; sc-40; Lot# J0220); AR (1:1000; sc-7305; Lot# J2920); PTEN (1:1000; sc-7974; Lot# 10420). The GAPDH antibody (1:5000; sc-32233; Lot# J2020) was used as an internal loading control. Membranes were washed and incubated with anti-mouse secondary antibody (1:2500; sc-2005). All the antibodies were purchased from Santa Cruz Biotechnology (Dallas, TX). The antigen-antibody reaction was visualized using an enhanced Chemiluminescence (ECL) assay (Bio-Rad; Hercules, CA, USA) and image the membrane using digital imager/chemidoc MP imaging system (Bio-Rad). A densitometry program using ImageJ (https://imagej.nih.gov/ij/), was used to quantify bands in the western blot and protein expression level displayed as ratio of each protein to the GAPDH protein level. The data are a representative of triplicate experiments.

### RNA extraction/Real-Time PCR

Cells were grown to 70% confluence and total RNA isolated using TRIzol reagent (Invitrogen) according to the instructions of the manufacturer. Total RNA (2 ug) was reverse transcribed to cDNA with the iScript cDNA synthesis kit (Bio-Rad) following the protocol of the manufacturer. Real-time quantitative RT-PCR was carried out for different gene amplicons using Taqman assays (Supplementary Table 1) in CFX96 real time PCR machine (Bio-Rad) and the following amplification at 35 cycles of 95^°^C for 5 s and 60^°^C for 30 s. The GAPDH or Keratin 18 Taqman assays was used as an endogenous control and RT-PCR analysis were done in triplicate.

### Wounding assay of scatter/migration

Prostate cancer cells were seeded at 0.25 × 10^6^/well in 6-wells plate and grown to confluence in complete medium and analyzed using a classical scratch wound method. Cells were gently scraped with a plastic tip. The medium was removed, and cells were washed twice with PBS. Complete medium was added, and cells were allowed to scatter/migrate into the area of clearing for a total of 48 hours and photomicrographs taken at 0, 24, and 48 hours.

### Cell cycle analysis

Cells (1 × 10^6^) were harvested by trypsinization, washed twice in PBS, and fixed in cold 70% ethanol for 18 hours at 4^°^C or −20^°^C. Fixed cells were washed in PBS once and incubated with propidium iodide/RNase staining buffer for 30 min at 37^°^C. Flow cytometry was performed by a BD FACSCalibur™ Flow Cytometer (BD Biosciences, San Jose, CA) and analyzed using FlowJo software.

### Statistical analysis

All experiments were repeated three times, and results are presented as the mean ± SD. Analyses of significance were performed using Student’s *t*-tests, Fisher test or one-way ANOVA. *P* < 0.05 was considered statistically significant.

## SUPPLEMENTARY MATERIALS


