# Supplementary material for: RASAL2 suppresses the proliferative and invasive ability of PC3 prostate cancer cells
Source: Oncotarget. 2021 Dec 21;12(26):2489–99. doi: 10.18632/oncotarget.28158 (PMC8711570; doi:10.18632/oncotarget.28158)
Supplement: Supplementary file 1 [file oncotarget-12-2489-s001.pdf]

RASAL2 suppresses the proliferative and invasive ability of PC3 prostate cancer cells

SUPPLEMENTARY MATERIALS

Supplementary Table 1: Gene abbreviation and list of primer sequences used in taqman RT-PCR assays

Gene Abbreviation	Forward Primer (5′–3′)	Reverse Primer (5′–3′)	Taqman (5′–3′)
RASAL2	GAGTACACCTACACCCAACAAA	CTCCATAGGCAGAATGGTGATAG	TCCGAATAGAAGGTCCTCCTGTCTTTCC
K-RAS	GGAGAAACCTGTCTCTTGGATATT	CTCATGTACTGGTCCCTCATTG	TCGACACAGCAGGTCAAGAGGAGTA
TNF-α	GAGACAGAAAGAGCGGGAAATA	ATTCACCTTCCAGGCATTCA	TTTCCCTGAGTGTCTTCTGTGTGCC
c-Myc	ACCACCAGCAGCGACTCTGA	TCCAGCAGAAGGTGATCCAGGCT	ACCTTTTGCCAGGAGCCTGCCTCT
CCR1	ACCAGCATCTACCTCCTGAACC	AGAGGATCTTACACATGGCATCAC	ACCTGCTCTTCCTGTTCACGCTTCC
CXCL5	GGTCCTTCGAGCTCCTTGTG	ATGAACTCCTTGCGTGGTCTG	TGCTGCTGCTGCTGCTGACGC
KERATIN 18	CCGAGAGGAGCTAGACAAGTA	ATCTCCAAGGACTGGACTGTA	ACTGTGGTGCTCTCCTCAATCTGC
GAPDH	GGTGTGAACCAT GAGAAGTATGA	GAGTCCTTCCACGATACCAAAG	AGATCATCAGCAATGCCTCCTGCA
